# “I rather talk on the phone”: Factors affecting compliance with outpatient visits during COVID-19 Pandemic

**DOI:** 10.1192/j.eurpsy.2022.1459

**Published:** 2022-09-01

**Authors:** F. Arain, N. Motamedi, N. Hassan, A. Zamiri, A. Rashid, M. Jennings, A. Sanchez-Lacay, P. Korenis

**Affiliations:** 1 BronxCare Health System Icahn School of Medicine at Mount Sinai, Child & Adolescent Psychiatry, Bronx, United States of America; 2 Bronx Care Health System-Affiliated with the Icahn School of Medicine at Mount Sinai, Psychiatry, New York, United States of America

## Abstract

**Introduction:**

The COVID-19 pandemic presented a global public-health crisis that demanded healthcare to adapt at an unprecedented pace. While challenging, it also created opportunities for the advancement of novel electronic-treatment-modalities. Telepsychiatry has emerged as an effective method to ensure continuity of care and ensure social distancing.^1^ Studies indicate that mental-health patients have higher rates of noncompliance to follow-up, ^1^ thus finding means to increase compliance is critical.

**Objectives:**

The objectives of this study are to determine the impact of telepsychiatry on compliance to follow-up and to identify numbers of psychiatric/medical emergency-room visits, most common contributing factors for admission, and compliance in terms of diagnosis.

**Methods:**

This IRB approved study is a retrospective chart-review, that aims to study children/adolescents (5-18 years) who presented to the Child&Adolescent-Psychiatry Outpatient-clinic from July-December 2020 and engaged in telepsychiatry, compared to a group of patients presented in July-December 2019-Pre-Covid19-Pandemic. A review of clinical characteristics including diagnosis, demographic information, medication, and treatment compliance will be compared as well as admissions to inpatient-psychiatry/emergency-room visits.

**Results:**

Our total sample (N=252) included patients from 2019-Pre-COVID19 (N=111) and 2020 Telehealth during COVID19-Pandemic (N=141). Our data analysis using SPSF and T-test has shown that Telehealth has significantly increased follow-up compliance (Two-tailed P-value=0.04); 2019-Pre-COVID outreach mean=0.06, 2020-Telehealth-during COVID outreach mean=0.02); significantly decreased ER/CPEP visits (P-value=0.02), and decreased In-patient-unit admissions (P-value=0.02).

**Conclusions:**

According to the presented study, the incorporation of telepsychiatry has increased the compliance to psychiatric-care in outpatient and decreased the emergency-room visits and inpatient admission. Sufficient resources and steps need to be taken to further strengthen telehealth services.

**Disclosure:**

No significant relationships.

